# A novel integrated training program for police pistol use across multiple operational scenarios: a randomized controlled trial of integrated psychological skill

**DOI:** 10.3389/fpsyg.2026.1806651

**Published:** 2026-03-20

**Authors:** Xiaoyue Liang, Chaoxin Ji, Yutong Wang, Lianzhong Cao

**Affiliations:** 1 Graduate School, Harbin Sport University, Harbin, China; 2 Department of Physical Education, Northeastern University, Shenyang, China; 3 Winter Olympic School, Harbin Sport University, Harbin, China

**Keywords:** autonomic regulation, heart rate variability, mindfulness training, police shooting, psychological skills training, resonance frequency breathing

## Abstract

This study investigated the effects of Resonance Frequency Breathing (RFB), Mindfulness Training (MT), and Combined Training (CT) on heart rate (HR), heart rate variability (HRV), mindfulness level, and pistol-shooting performance among police academy cadets. Eighty cadets were randomly assigned to four groups (*n* = 20 each), including three experimental groups and a Control Group. HR, HRV frequency-domain indices [low frequency (LF), high frequency (HF), LF/HF ratio], total mindfulness questionnaire scores, and shooting performance in the Static, Rapid, and Tactical Shooting Tasks were measured before and after the intervention. All three interventions significantly reduced HR (RFB *p* < 0.05; MT *p* < 0.01; CT *p* < 0.001) and increased HRV indices (*p* < 0.001). Mindfulness scores improved significantly in the MT and CT groups (*p* < 0.001), whereas the RFB group showed a slight decrease (*p* < 0.05). A significant group × time interaction effect was observed for mindfulness (η*_p_*^2^ = 0.842). Shooting performance improved significantly in all experimental groups (*p* < 0.001), with large between-group effect sizes observed in Static Shooting (η*_p_*^2^= 0.685), Rapid Shooting (η*_p_*^2^ = 0.585), and Tactical Shooting (η*_p_*^2^ = 0.518). The CT group demonstrated the greatest performance improvements, particularly in complex shooting tasks. The control group showed a minor increase in overall shooting score (*p* < 0.05) but no significant changes in individual tasks (*p* > 0.05). These results suggest that CT effectively enhances autonomic regulation and psychological readiness, leading to better shooting performance in police cadets.

## 1 Introduction

In high-risk law enforcement and emergency response settings, police pistol shooting is a high-precision perceptual-motor task that requires exceptional motor control and psychological regulation under pressure. Unlike standardized operations in competitive shooting or training, police shooting often occurs under severe time constraints, high uncertainty, and significant threats to life. In these conditions, officers must execute a continuous sequence of perception, decision-making, and motor actions within a minimal timeframe, while managing intense physiological arousal and psychological stress. Under such pressure, imbalances in the autonomic nervous system (ANS) activity, limited attentional resources, and reduced emotion regulation capacity can substantially impair motor control stability and shooting accuracy, adversely affecting overall performance (Zhao et al., 2024). Therefore, from a psychology perspective, investigating approaches to enhance individuals’ self-regulation capacity under conditions of high stress and high task complexity has become a key issue of shared concern in the fields of high-risk operational task training and applied psychology.

In recent years, psychophysiological regulation-based training approaches have gained increasing attention in research on high-precision motor performance. Among these, Resonance Frequency Breathing (RFB) is a physiological intervention grounded in heart rate variability (HRV) biofeedback, with theoretical underpinnings in respiratory-cardiac coupling and baroreflex models. By guiding individuals to breathe rhythmically near their personal resonance frequency (≈0.1 Hz), RFB increases the amplitude of respiratory sinus arrhythmia, enhances baroreflex sensitivity, and elevates parasympathetic activity, thereby improving autonomic nervous system (ANS) balance (Lehrer et al., 2000; Lehrer and Gevirtz, 2014). Experimental studies indicate that low-frequency paced breathing significantly elevates HRV and enhances autonomic regulation in both healthy adults and populations exposed to varying psychological demands (Sakakibara et al., 2020; Leganes-Fonteneau et al., 2021; Bahameish and Stockman, 2024). Psychophysiological evidence further suggests that RFB strengthens vagal afferent signaling and baroreflex functioning, potentially influencing psychological regulation by reducing interoceptive prediction error and promoting awareness of bodily signals (Leganes-Fonteneau et al., 2021). At the applied level, RFB has been integrated into studies of competitive sports and high-demand operational tasks, demonstrating reductions in stress responses alongside enhanced physiological stability and motor or cognitive control. For instance, in basketball-specific performance contexts, RFB has been found to improve athletes’ physiological regulation and performance stability (Göçmen et al., 2023). In non-sport domains, RFB has also been shown to support executive functions, including working memory and inhibitory control, through HRV augmentation (Bahameish and Stockman, 2024; Spalding et al., 2025). Neuroimaging studies further indicate that controlled deep breathing modulates both HRV indices and activity in brain regions linked to emotion regulation and cognitive control, corroborating the role of respiration-driven brain–heart coupling in stress regulation (Huber et al., 2025).

In contrast to the physiologically oriented nature of RFB, Mindfulness Training (MT) targets attentional and awareness processes. It promotes nonjudgmental engagement with present-moment experience, reducing emotional reactivity and cognitive interference while strengthening attentional control and psychological stability (Kabat-Zinn, 2003). In recent years, MT has been widely implemented in psychological interventions across competitive sport, military training, and other high-stress occupational settings. Results from systematic reviews and meta-analyses indicate that mindfulness-based interventions demonstrate moderate effect sizes in improving athletes’ anxiety levels, attentional focus, and overall sport performance, and these effects have been validated across multiple sports disciplines (Wang et al., 2023; Reinebo et al., 2024). In military and quasi-military contexts, MT has been shown to contribute to the regulation of ANS activity, improve emotion regulation capacity, and enhance individuals’ psychological adaptability under high-stress training conditions (Lee et al., 2025; Jha et al., 2025). From a performance psychology perspective, MT facilitates improvements in consistency and stability during the execution of high-load and complex tasks by optimizing attentional resource allocation and reducing anxiety-related interference (Li et al., 2025).

Although both RFB and MT have been shown to improve physiological or psychological regulation capacities across different contexts, direct experimental evidence comparing whether the two approaches exert complementary effects on motor performance tasks under high pressure and varying levels of task complexity remains limited. In particular, in shooting tasks that require rapid responses, fine motor control, and stress regulation, it remains unclear whether psychological skills training focused on physiological regulation and cognitive-attentional regulation can interact synergistically to further enhance performance stability.

Taken together, existing literature suggests that physiological regulation and attentional regulation represent two complementary pathways through which psychological skills training may influence performance under stress. RFB primarily targets bottom–up physiological regulation by stabilizing autonomic activity through respiratory–cardiac coupling and enhanced baroreflex sensitivity, thereby supporting more stable physiological arousal during demanding tasks. In contrast, mindfulness training primarily operates through top–down cognitive mechanisms, improving attentional control, reducing emotional reactivity, and promoting greater psychological stability during task execution. From a performance perspective, complex operational tasks such as police shooting require the coordinated functioning of both physiological regulation and cognitive–attentional control systems. However, previous research has largely examined these approaches independently, and empirical evidence comparing their relative or combined effects on high-pressure perceptual–motor tasks remains limited. Clarifying whether physiological regulation and mindfulness-based attentional training exert complementary or synergistic effects may therefore provide important insights for optimizing psychological skills training in high-risk operational contexts.

Based on these considerations, the present study was grounded in the practical training demands of law enforcement and recruited police academy cadets as participants, adopting a randomized controlled trial design to systematically compare the effects of RFB, MT, and their CT on multi-context pistol-shooting performance. By establishing an RFB group, an MT group, a CT group, and a Control Group that received routine instructional training, the four-week intervention comprehensively examined the effects of different training approaches on cadets’ autonomic regulation, psychological regulation capacity, and shooting performance. Shooting performance assessment encompassed multiple task contexts, including the Static Shooting Task (SST), Rapid Shooting Task (RST), and Tactical Shooting Task (TST), to test the differential effects of psychological skills training under conditions of increasing task complexity and stress.

Based on theories of autonomic regulation, mindfulness-based attentional control, and empirical findings from research on motor performance under high pressure, the present study proposed the following hypotheses: H1. Compared with the Control Group, participants receiving RFB, MT, and CT will exhibit superior autonomic regulation following the intervention, as reflected by reduced resting HR and significant improvements in HRV frequency-domain indices [low frequency (LF), high frequency (HF), and LF/HF ratio]. Among the three interventions, CT is expected to produce the greatest magnitude of improvement in psychophysiological regulation indices. H2. Compared with the Control Group, participants in the MT group and the CT group will demonstrate significantly higher levels of mindfulness following the intervention, whereas the direct effect of RFB on mindfulness levels will be relatively limited. H3. Compared with the Control Group, all three training approaches will significantly enhance shooting performance in the SST, RST, and TST. Among them, CT will demonstrate a more pronounced advantage in tasks conducted under conditions of greater complexity and pressure, such as the RST and the TST.

## 2 Methods

### 2.1 Participants

The sample for the present study was drawn from a convenience sample of cadets enrolled in regular coursework at a police academy. The study was implemented within the framework of an established police weapon-use training curriculum, and the sample size was constrained by class enrollment and training conditions. All participants had completed basic police weapon-use courses and possessed fundamental pistol handling skills. Prior to the commencement of the experiment, all participants were fully informed of the study purpose, process and potential risks before the experiment began and signed a written informed consent form. Participants could withdraw at any time without consequences for coursework participation or performance evaluation. The study was approved by *the Ethics Committee of Harbin Sport University* (Approval No. 2025350) and conducted in accordance with the 2013 Declaration of Helsinki.

**Figure 1:**
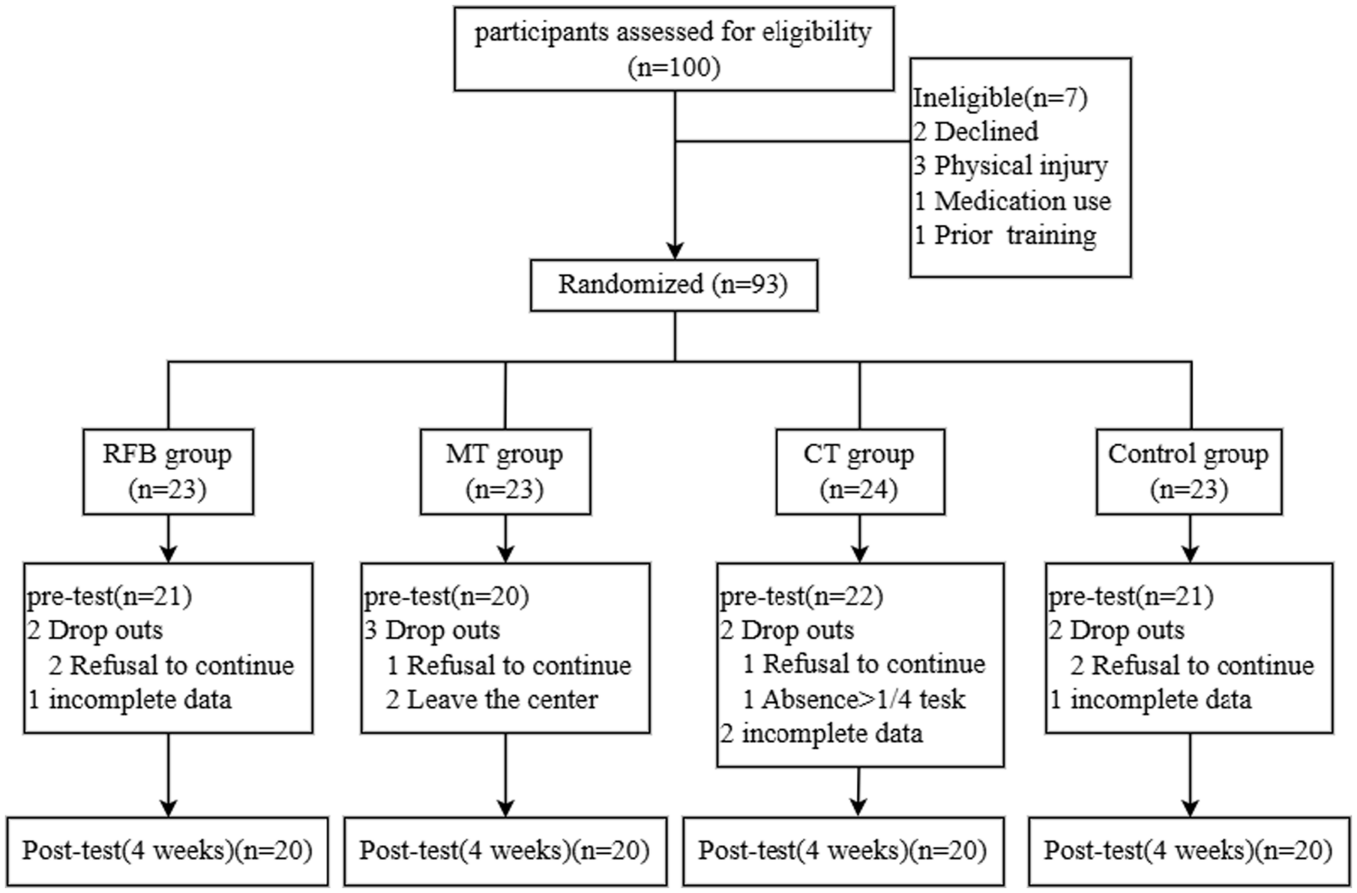
Flowchart of participant enrollment and allocation.

An *a priori* power analysis was conducted in G*Power 3.1 (Faul et al., 2007). Assuming a medium effect size of 0.25, an alpha of 0.05, a statistical power of 0.95, four groups, and two repeated measurements, the required minimum sample size was 76. Considering a 10% dropout rate and the possibility of subject attrition, a total of 100 participants majoring in Criminal Investigation were initially recruited and screened. The final sample included 80 participants (20 per group) (Figure 1), providing adequate statistical power (=0.968). Participant demographics are presented in Table 1, with no baseline differences observed across groups.

**Table 1:** Participants characteristics (M ± SD).

Characteristics	RFB group (*n* = 20)	MT group (*n* = 20)	CT group (*n* = 20)	Control group (*n* = 20)	*p*-value
Gender (female %)	2(10%)	2(10%)	2(10%)	2(10%)	1.000
Age (year)	20.15 ± 0.32	20.36 ± 0.47	20.27 ± 0.36	20.30 ± 0.52	0.773
Height (cm)	179.34 ± 6.21	178.87 ± 5.12	179.32 ± 5.03	178.21 ± 6.14	0.540
Weight (kg)	73.56 ± 6.43	74.50 ± 5.98	72.69 ± 5.64	71.53 ± 6.21	0.955
Dominant eye visual acuity (decimal)	4.90 ± 0.13	4.91 ± 0.12	4.91 ± 0.17	4.90 ± 0.15	0.795

Values expressed as M ± SD. n, sample; M, Mean; SD, Standard Deviation. Visual acuity was measured using a logarithmic visual acuity chart commonly used in China (logMAR-based) and reported in decimal form.

### 2.2 Experimental design

The study used a randomized controlled trial design with standardized intervention and measurement procedures to reduce systematic bias and strengthen internal validity. Its purpose was to compare the effects of different psychological skills training programs on multi-context pistol-shooting performance among police academy cadets. Participants were assigned to four conditions: an RFB group, an MT group, a CT group, and a non-intervention control group. An independent statistician generated the computer-based randomization sequence in Microsoft Excel. Group assignments were concealed using sequentially numbered opaque sealed envelopes. After baseline testing and written informed consent were completed, the research coordinator opened the next envelope to determine allocation. Because of the characteristics of the interventions, instructors were unavoidably unblinded after allocation. However, throughout the trial, outcome assessors and data analysts remained blinded to group allocation. All interventions were implemented within the same training period, and the study procedures followed the principle of integrating experimental control with operational realism to enhance both internal and applied validity of the study findings.

### 2.3 Interventions

**Figure 2:**
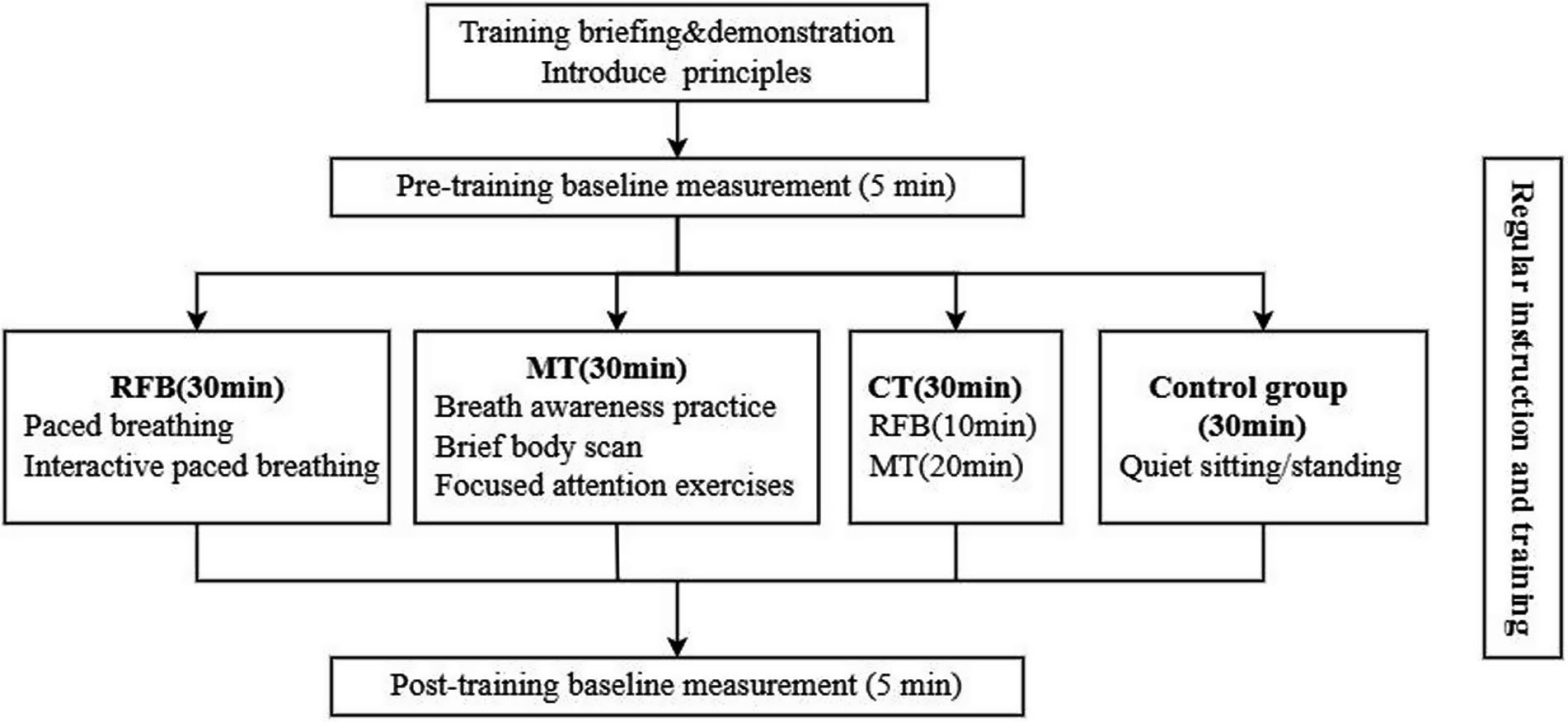
Study intervention flowchart.

All intervention training sessions were conducted within the regular instructional cycle of the police academy. The intervention period lasted 4 weeks, with training conducted 5 times per week. Each training session was scheduled to last approximately 40 min. Training frequency, total session duration, and intervention period were kept identical across all experimental groups in order to control for potential confounding effects associated with differences in training dosage (Figure 2).

Within the intervention design, the core intervention time of each training session was defined as the structured training period devoted to the implementation of the primary training content (≈ 30 min), while the remaining time was mainly allocated to pre- and post-training physiological measurements and necessary procedural preparation and instructions. The revised Five Facet Mindfulness Questionnaire (FFMQ) and shooting performance indices were used as study-level assessment tools and were administered before and after the 4-week intervention, and were not included in repeated measurements within individual training sessions. This design ensured consistency in total training exposure across groups while allowing each intervention approach to exert its specific psychological and physiological regulation mechanisms within an identical core training window. All interventions were conducted by trained research staff or instructors in a consistent training environment following standardized protocols.

#### 2.3.1 Resonance frequency breathing training group

The RFB training protocol was adapted from the HRV biofeedback program described by Lehrer et al. (2000). To ensure feasibility and standardized delivery in a law enforcement training context, participants maintained a uniform breathing rhythm of roughly 6 breaths per minute, which approximates the group-average resonance frequency (Lehrer and Gevirtz, 2014).

Each session included a 5-min pre-training baseline, a 10-min rhythmic-breathing phase, a 20-min cue-guided breathing-maintenance phase, and a 5-min post-training baseline. During training, participants practiced slow and even diaphragmatic breathing in either a seated or standing position while following rhythmic cues. Breathing timing was guided by visual or auditory signals generated by a custom program indicating inhalation and exhalation. Participants used a nasal inhalation and oral exhalation pattern, with the exhalation phase slightly longer than the inhalation phase to promote parasympathetic activation. The cues supported the stable maintenance of rhythmic breathing rather than the dynamic adjustment of breathing frequency based on real-time physiological indicators. No shooting tasks or additional psychological guidance were included to maintain methodological isolation of the intervention pathway.

Before the first session, research staff provided standardized instruction on the theoretical principles and operational procedures of RFB training, followed by a demonstration practice. From the second session onward, participants practiced independently following the predefined protocol.

#### 2.3.2 Mindfulness training group

The MT intervention was guided by a mindfulness-based cognitive training framework. Participants were guided to attend to present-moment experiences with a nonjudgmental attitude, including bodily sensations, breathing process, and immediate mental activities (Tang et al., 2015). This approach was intended to strengthen attentional stability and emotion regulation while reducing attention fluctuations induced by emotional and cognitive interference under high-pressure conditions (Prakash, 2021; Liu et al., 2023).

Each session lasted about 40 min, with nearly 30 min devoted to core MT practice. Five-minute resting baselines were recorded before and after each session to collect physiological indices for group comparisons and evaluation of intervention effects. No physiological feedback was provided during MT, and no external pacing or rhythm control was imposed. Training was delivered through guided audio instruction combined with self-directed practice (Sparacio et al., 2024). Content included breath awareness, brief body-scan exercises, and short focused-attention practices, emphasizing awareness rather than deliberate control, and avoiding reducing MT to relaxation or breathing-regulation exercises.

To enhance the contextual relevance of the training for police shooting tasks while avoiding the introduction of shooting-technique practice during the intervention phase, MT was conducted under dry-fire conditions. Participants adopted a simulated firearm-holding posture and were guided to observe attentional fluctuations, emotional responses, and decision-related cognitive activities without performing shooting actions or receiving technical correction.

#### 2.3.3 Combined training group

Using the same training cycle and session structure, the CT group followed an integrated protocol combining RFB and MT to examine their potential synergistic effects on autonomic regulation and attention-emotion regulation mechanisms. Five-minute resting baselines were recorded before and after each session. The core intervention phase occurred between the two baselines and lasted approximately 30 min, comprising about 10 min of RFB followed by 20 min of MT. Training emphasized continuity and consistency between breathing rhythm regulation and awareness-oriented practices, and no shooting tasks or additional psychological guidance were introduced.

#### 2.3.4 Control group

The Control group did not receive any psychological or physiological regulation intervention. The training cycle, session structure, and measurement procedures were identical for all experimental groups. In each session, participants remained seated or standing quietly under natural breathing conditions, without rhythmic cues or attentional guidance. The Control group participated only in the routine police academy instructional program, which matched that of the experimental groups but excluded the intervention training used in this study. Shooting test scenarios, testing procedures, and performance evaluation criteria were kept consistent across all groups.

### 2.4 Shooting tasks and experimental scenarios

To evaluate the effects of different psychological interventions on police shooting performance across varying task demands and contextual complexity, three shooting tasks were used: SST, RST, and TST. These tasks represented a progressive increase in operational demands, time pressure, and cognitive load (Simas et al., 2022), thereby forming a multi-context assessment system spanning stable to highly complex conditions. All tests were conducted at the police academy shooting center using standard 9-mm QSZ92-9G semi-automatic pistols, 9-mm DAP92A service ammunition, and standard police targets. Each magazine was loaded with five rounds. All tests were supervised by the same certified instructors to ensure safety and procedural consistency. Shooting procedures and performance criteria were identical across groups. The three tasks differed in shooting distance, target type, time limits, and operational requirements, creating distinct levels of situational load.

#### 2.4.1 Static shooting task

The SST assessed basic shooting accuracy and motor control under low contextual interference. During the test, participants stood with two hands unsupported, facing fixed targets at the designated firing position. The shooting distance was 15 m. A 500 × 500 mm chest-ring target with 50 mm ring spacing was used. The target was exposed for 6 s per trial, during which one shot was fired, for a total of five shots. The timing was intended to standardize shooting rhythm rather than introduce speed demands or time pressure.

#### 2.4.2 Rapid shooting task

The RST added explicit time constraints and posture transitions to the SST to examine attentional allocation, motor transitions, and shooting efficiency under moderate time pressure. Shooting distance was 7 m, using a 1,000 × 500 mm half-body target. Total target exposure time was 22 s. Within this interval, participants completed 10 shots, performing five in a standing posture and five in a kneeling posture. The transition from standing to kneeling was guided by standardized verbal instructions.

#### 2.4.3 Tactical shooting task

The TST was designed to simulate complex law enforcement shooting scenarios that more closely resemble real-world police operations by incorporating cover use, multiple-target engagement, and situational judgment, and time constraints. During the test, participants completed the task within a cover environment composed of terrain features including wall corners, vehicle bodies, and soil mounds. The target setup included one half-body target, one head target (200 × 300 mm), and one hostage-taking target (1,700 × 500 mm), with an effective hit area of 200 × 300 mm on the hostage target. The target presentation duration was 35 s. Participants were required to complete two magazine changes within the prescribed time and to engage each of the three target types, performing 5 shooting actions per target type, for a total of 15 rounds. Target transitions were performed in conjunction with changes in cover position during the shooting process.

#### 2.4.4 Control of shooting task order and performance validity

The three shooting tasks were performed sequentially in the order of the SST, RST, and TST, in order to avoid interference of high-load tasks with basic shooting performance. In the RST and TST, to ensure the standardization and comparability of performance evaluation, Valid shooting performance was uniformly defined as only those specified shooting actions that were completed within the prescribed time and in accordance with the task structure requirements. Shots that missed the target, exceeded the prescribed number of shots, or were completed outside the time limit were not included in performance scoring. Because the SST physically controlled the shooting rhythm through target presentation time, there was no possibility of exceeding the prescribed number of shots or time limits; therefore, only hits and misses were recorded as performance indicators. All shooting tasks were administered once before the intervention and once after the completion of the 4-week intervention, to evaluate changes in shooting performance across different training conditions.

### 2.5 Outcome measures and data collection

#### 2.5.1 Heart rate variability measures

HRV was used as the primary physiological outcome measure in this study to reflect changes in autonomic nervous system regulation. HRV is widely applied to evaluate individuals’ physiological adaptive capacity in stress-related, emotion regulation, and self-control–related contexts, and is particularly well suited for psychophysiological research involving high-stress occupational populations.

With respect to the selection of HRV analysis methods, commonly used indices mainly include time-domain measures and frequency-domain measures. Time-domain indices are derived from the statistical characteristics of RR intervals and reflect overall variability; however, their ability to differentiate specific autonomic regulatory mechanisms is relatively limited. In contrast, frequency-domain analysis decomposes RR variability into spectral components across frequency bands and is more sensitive to short-term autonomic regulation changes (Besson et al., 2025). It is therefore widely used in psychophysiological and sport science research.

It should be noted that the physiological interpretation of frequency-domain HRV indices remains debated, particularly regarding LF and the LF/HF ratio. LF should not be treated as a direct marker of cardiac sympathetic activity, as its variation does not consistently correspond to sympathetic measures derived from positron emission tomography or norepinephrine spillover. Instead, LF is largely co-regulated by vagal activity and baroreflex mechanisms (Amekran et al., 2024; Li et al., 2026). Accordingly, neither absolute nor normalized LF, nor the LF/HF ratio, should be simplistically equated with sympathetic function or interpreted as a direct index of sympathovagal balance (Quigley et al., 2024). Although HF is also influenced by respiration, heart rate, and certain non-autonomic factors, under resting conditions with stable breathing it primarily reflects respiratory sinus arrhythmia and is therefore considered a relatively reliable index of cardiac vagal modulation. Changes in the LF/HF ratio may indicate the direction of autonomic regulatory shifts, but its numerical value cannot quantify the relative strength of sympathetic and vagal activity, nor autonomic regulatory capacity. On this basis, the present study emphasizes overall HRV levels and spectral trend patterns, rather than attributing LF, HF, or the LF/HF single linear interpretations of specific autonomic neural functions.

HR signals were recorded using the Polar H10 chest-strap monitor (Polar Electro Oy, Finland), which records data based on ECG-based RR interval detection. The device has been widely used in sport science and psychophysiological research and has demonstrated high reliability and validity for HRV assessment in both laboratory and applied settings.

HRV was recorded in a quiet indoor setting with participants seated at rest. Participants were instructed to remain still, breathe naturally, and refrain from speaking or unnecessary movement. Each recording lasted 5 min, in line with international recommendations for short-term HRV assessment. To control for circadian influences, pre- and post-intervention measurements were conducted at the same time of day. RR intervals were exported by Kubios HRV software (Standard version, Finland) for analysis (Tarvainen et al., 2014). Recordings were visually inspected, and artifacts were corrected using the software’s standard automated correction algorithm. Recordings with more than 5% corrected RR intervals were considered to have insufficient signal quality and were excluded from analysis. In the present dataset, all recordings met this criterion and were therefore retained for statistical analysis.

Frequency-domain analysis used spectral analysis based on the Fast Fourier Transform (FFT), with LF (0.04–0.15 Hz) and HF (0.15–0.40 Hz) expressed in absolute units (ms^2^). The LF/HF ratio was derived from normalized spectral components after exclusion of very-low-frequency power. For each participant, indices were averaged across the recording period for statistical analysis. Total power served only as an intermediate normalization variable and was not reported. All procedures followed current international consensus guidelines for HRV measurement and reporting.

#### 2.5.2 Psychological measures

Mindfulness was assessed using the Five-Facet Mindfulness Questionnaire (FFMQ) (Baer et al., 2006; Meng et al., 2020). The questionnaire comprises 39 items across five dimensions: observing, describing, acting with awareness, non-judging, and non-reactivity. Items are rated on a 5-point Likert scale, with higher scores reflecting greater levels of the corresponding mindfulness characteristics.

Given the high situational stress, time pressure, and decision uncertainty inherent in police shooting tasks, the wording of certain original items did not fully correspond to the practical context of police shooting training. To enhance contextual applicability and content validity, they were reworded while preserving the original factorial structure and dimensional framework of the FFMQ. Revisions were informed by prior literature, the practical demands of police shooting training, and expert consultation, with particular attention to maintaining semantic correspondence with attentional control, emotional awareness, and behavioral responses relevant to shooting performance. For example, an item from the observing dimension reads: “I pay attention to physical sensations such as breathing or muscle tension while preparing to shoot,” and an item from the acting-with-awareness dimension reads: “When performing a shooting task, I focus fully on the current action rather than operating automatically.”

The adapted FFMQ was piloted in 20 students with shooting experience, with a response rate of 100%. Internal consistency of all dimensions exceeded 0.7. In the present study sample, Cronbach’s α coefficients for the five dimensions at pre-intervention were 0.831, 0.703, 0.729, 0.793, and 0.742, and at post-intervention were 0.852, 0.729, 0.843, 0.855, and 0.862, indicating good internal consistency. It should be noted that only the wording of the scale items was contextually adjusted, and the structure and dimensions remained unchanged. Therefore, reliability was assessed using Cronbach’s α, and content validity was confirmed through expert review. Given the limited sample size of the pilot survey (*n* = 20), it was not sufficient to conduct factor analysis for construct validity.

Given the study’s overall design, the FFMQ was not included in repeated measurements during individual training sessions and was administered only once before the start of the intervention and once after the 4-week intervention period. Participants completed the questionnaire independently in a quiet setting. Standardized instructions were provided by the researchers to ensure consistency in administration and data collection. The resulting psychological measures were used to examine intervention-related changes in mindfulness traits across the study period.

#### 2.5.3 Shooting performance measures

Shooting performance served as the primary behavioral outcome measure in this study, reflecting intervention effects on police shooting ability across tasks of differing complexity. Scores were derived from the SST, RST, and TST. Testing procedures and scoring criteria followed the official police firearms training standards, with detailed task parameters described in section 2.5.

All scores were recorded by certified instructors using standardized criteria. SST performance was evaluated primarily by hit accuracy and ring scores. For the RST and TST, effective scores were calculated according to task-specific structural requirements in addition to hit outcomes. Each shooting task was administered once pre-intervention and once after the 4-week intervention to assess both within-group and between-group performance changes.

### 2.6 Statistical analysis

Data were organized using Microsoft Excel 2021 and analyzed with SPSS 25.0 (IBM Corp., Armonk, NY, United States). For each group, pre-intervention (pre), post-intervention (post), and change scores (post-pre) were summarized as mean ± standard deviation (M ± SD).

Given the randomized controlled pre-post design of the study and the primary interest in between-group differences in training-related changes under different intervention conditions, the main inferential analyses for physiological measures (HR and HRV) and shooting performance were conducted on the change scores (Δ = post−pre).

Within-group pre-post differences were analyzed using paired-sample *t*-tests. All statistical tests were two-tailed, with the significance level set at α = 0.05. Between-group differences were first assessed for homogeneity of variance using Levene’s test. As the change scores for the SST, RST, and TST violated the assumption of homogeneity of variance, overall between-group differences were analyzed using Welch’s one-way analysis of variance (Welch ANOVA), followed by pairwise comparisons with the Games-Howell *post-hoc* test to control for the impact of unequal variances on significance levels. Between-group effect sizes were reported as partial eta squared (η*_p_*^2^). The use of change scores helps reduce the influence of baseline individual differences and provides more robust statistical inference for comparing changes across intervention conditions, especially with a relatively small sample size and unequal variances.

For mindfulness outcomes, because repeated measurements were collected at different time points and baseline equivalence had been confirmed, a mixed-design (group × time) repeated-measures analysis of variance was conducted to directly examine intervention-specific time effects as well as group-by-time interactions. Tables 2–4 present descriptive statistics and corresponding significance tests for HRV, psychological measures, and shooting performance across groups.

**Table 2:** Descriptive statistics of heart rate variability indices before and after intervention across groups (M ± SD).

Group	Time	HR (beats/min)	LF (ms^2^)	HF (ms^2^)	LF/HF
RFB group (*n* = 20)	pre-test	77.13 ± 5.30	235.71 ± 59.93	87.43 ± 30.66	5.09 ± 1.54
	post-test	73.74 ± 1.70*	1226.39 ± 175.59***	130.72 ± 19.78***	23.47 ± 3.03***
MT group (*n* = 20)	pre-test	76.57 ± 4.33	216.35 ± 55.50	59.23 ± 21.90	7.37 ± 0.91
	post-test	74.03 ± 1.69**	803.29 ± 83.96***	110.99 ± 13.27***	19.12 ± 2.42***
CT group (*n* = 20)	pre-test	78.01 ± 5.26	280.49 ± 58.46	99.51 ± 25.51	6.20 ± 0.72
	post-test	73.23 ± 2.23***	1646.86 ± 205.95***	168.63 ± 18.85***	31.83 ± 3.90***
Control group (*n* = 20)	pre-test	76.50 ± 3.67	178.47 ± 32.85	34.54 ± 8.63	9.48 ± 1.30
	post-test	75.25 ± 0.76	221.17 ± 18.75***	48.76 ± 3.34***	10.01 ± 0.66

Values expressed as M ± SD. HR, heart rate; HRV, heart rate variability; LF, low-frequency power; HF, high-frequency power; LF/HF, ratio of low- to high-frequency power. RFB, resonance frequency breathing; MT, mindfulness training; CT, combined training. *p < 0.05, **p < 0.01, ***p < 0.001 vs. pre.

**Table 3:** Descriptive statistics of the total FFMQ scores under different intervention conditions (M ± SD).

Time	RFB group (*n* = 20)	MT group (*n*-20)	CT group (*n* = 20)	Control group (*n* = 20)
pre-test	115.35 ± 5.10	115.25 ± 4.64	115.95 ± 3.36	117.95 ± 3.78
post-test	113.10 ± 4.88*	131.85 ± 7.42***	135.55 ± 6.17***	116.20 ± 3.55*

*p < 0.05, **p < 0.01, ***p < 0.001 vs. pre.

**Table 4:** Descriptive statistics of shooting performance across intervention groups in different shooting tasks (M ± SD).

Performance	RFB group (*n* = 20)	MT group (*n* = 20)	CT group (*n* = 20)	Control group (*n* = 20)
Total score (pre-test)	231.10 ± 17.27	230.55 ± 17.64	231.90 ± 13.75	231.75 ± 14.00
Total score (post-test)	264.65 ± 11.55**	260.00 ± 10.31**	278.10 ± 6.40**	233.00 ± 14.20*
ΔSST. score	7.25 ± 2.61***	6.65 ± 3.08***	9.75 ± 2.40***	0.25 ± 1.37
ΔPST. score	12.15 ± 4.74***	8.75 ± 4.46***	15.20 ± 7.03***	0.30 ± 1.17
ΔTST. score	14.15 ± 8.65***	14.05 ± 8.29***	21.25 ± 8.80***	0.70 ± 1.03
Δtotal score	33.55 ± 9.12***	29.45 ± 11.25***	46.20 ± 14.86***	1.25 ± 2.22

SST, static shooting task; PST, rapid shooting task; TST, tactical shooting task. The total score represents the sum of SST, PST, and TST. The maximum possible scores are 50 for SST, 100 for PST, and 150 for TST, with a total maximum score of 300. ΔRepresents the change score (post−pre) for each shooting task. *p < 0.05, **p < 0.01, ***p < 0.001 vs. pre.

## 3 Results

### 3.1 Effects of training interventions on HR and frequency-domain HRV measures in police cadets

After the intervention, the mean HR of participants in all experimental groups decreased compared with pre-intervention values (Table 2). Significant reductions were observed in the RFB group (*p* < 0.05) and the MT group (*p* < 0.01), while the CT group showed a highly significant decrease (*p* < 0.001). In contrast, no significant change in HR was observed in the control group (*p* = 0.170). Regarding frequency-domain HRV indices, LF, HF, and the LF/HF ratio were all significantly higher after the intervention than before in each experimental group, with all differences reaching a highly significant level (*p* < 0.001). In the control group, statistically significant pre–post differences were also observed for LF and HF (*p* < 0.001); however, no significant change was found in the LF/HF ratio (*p* = 0.186).

**Figure 3:**
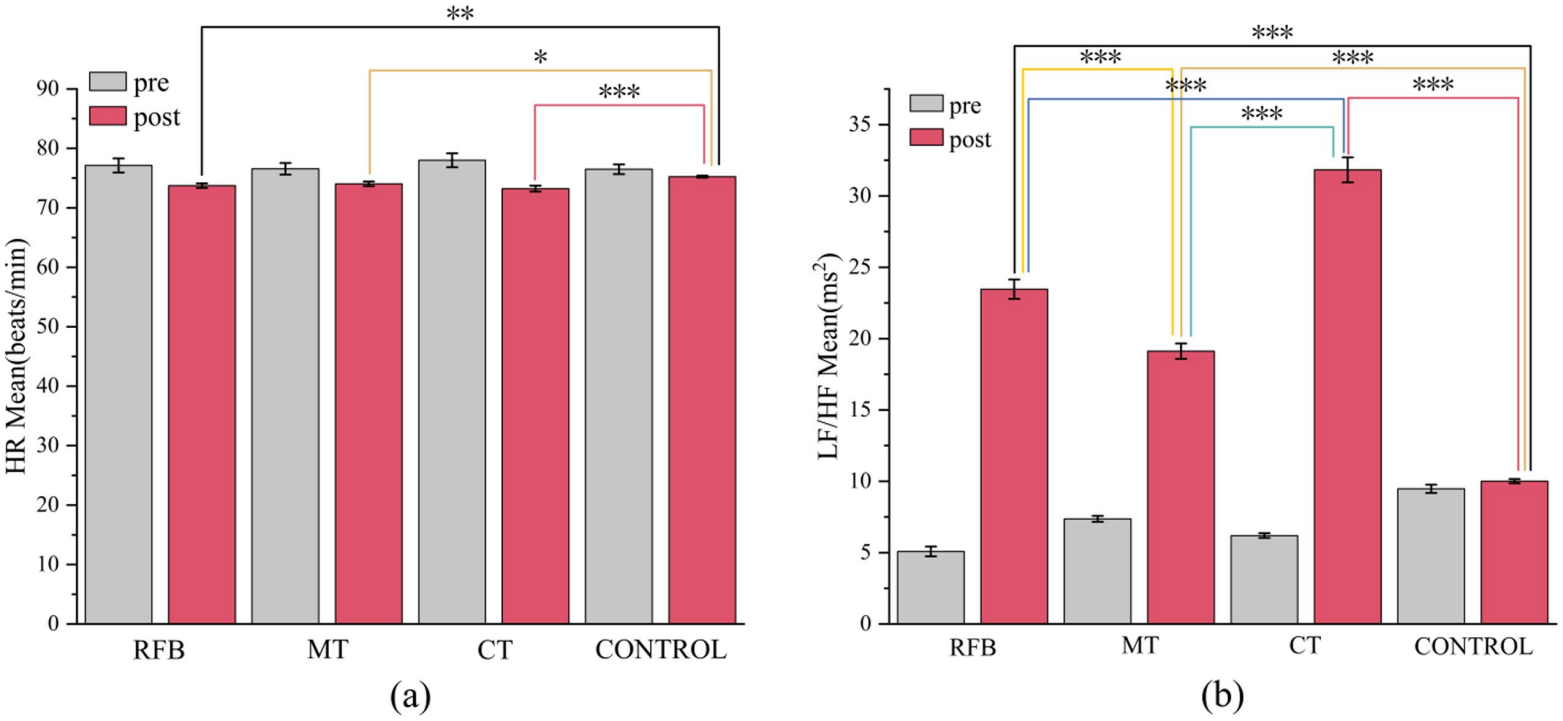
Pre- and post-intervention comparisons of HR and LF/HF ratio across training groups. **(a)** Mean HR values before and after the intervention for each group. **(b)** Mean LF/HF ratio before and after the intervention for each group. Gray bars represent pre-test values, and pink bars represent post-test values. Error bars indicate standard errors of the mean (M ± SE). Brackets and asterisks denote statistically significant between-group differences: *p <0.05**, *p* < 0.01, *** *p* < 0.001.

**Figure 4:**
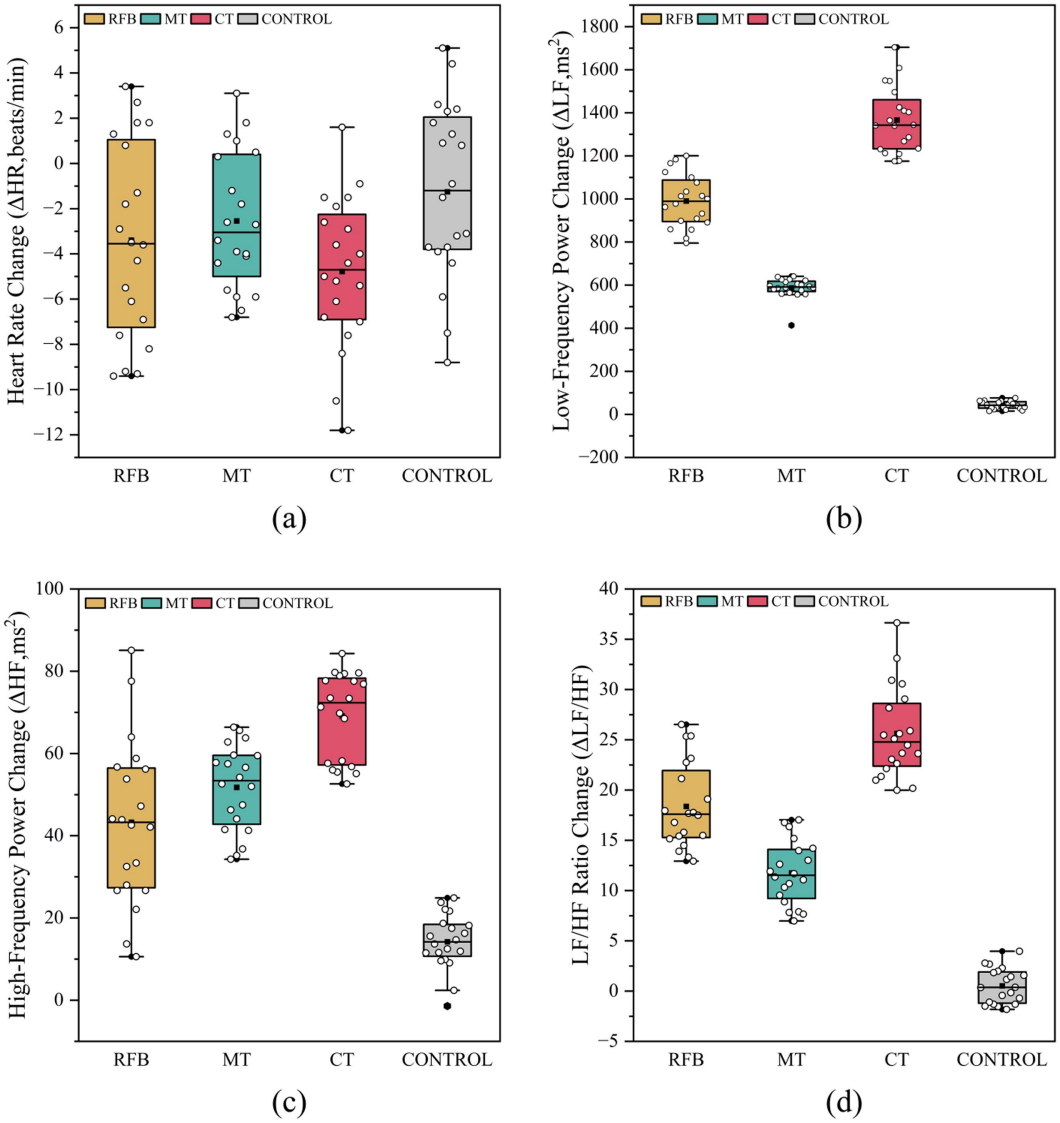
Changes in heart rate and frequency-domain heart rate variability indices following different training interventions. Distribution of pre–post changes (Δ) in heart rate (HR) and frequency-domain heart rate variability (HRV) indices across the RFB, MT, CT, and control groups. **(a)** Change in heart rate (ΔHR). **(b)** Change in low-frequency power (ΔLF). **(c)** Change in high-frequency power (ΔHF). **(d)** Change in LF/HF ratio (ΔLF/HF). Boxes represent the interquartile range (IQR) with the median indicated by the horizontal line; whiskers denote the range. White circular markers represent individual observations, black square markers indicate group means, and black hexagonal markers denote outliers.

Welch ANOVA conducted on ΔHR, ΔLF, ΔHF, and ΔLF/HF revealed significant between-group differences in △ HR [*F*(3, 41.878) = 3.323, *p* = 0.029, η*_p_*^2^ = 0.114], with the CT group showing a greater reduction in heart rate than the control group (*p* = 0.020). Between-group differences were highly significant for ΔLF, ΔHF, and ΔLF/HF [*F*(3, 34.638) = 1465.902; *F*(3, 40.239) = 147.396; *F*(3, 39.000) = 260.956; all *p* <0.001, η*_p_*^2^ = 0.992, 0.918, and 0.953, respectively]. *Post hoc* analyses indicated that the CT group exhibited the largest increases in ΔLF, ΔHF, and ΔLF/HF (all *p* < 0.001). In addition, the increases in these indices in the RFB and MT groups were significantly greater than those in the control group (*p* < 0.001). Furthermore, the RFB group showed significantly greater increases in ΔLF and ΔLF/HF than the MT group (*p* < 0.001). As shown in Figure 3 Pre- and post-intervention comparisons of HR and LF/HF ratio, post-intervention changes in HR and LF/HF differed across experimental groups, with the largest magnitude of change observed in the CT group. As shown in Figure 4, compared with the other groups, the CT group exhibited greater magnitudes of change in HR and HRV frequency-domain indices, whereas the control group showed relatively small overall changes.

### 3.2 Effects of training interventions on psychological measures in police cadets

Descriptive statistics of total mindfulness scores across groups under different training conditions are presented in Table 3. The total mindfulness score ranged from 39 to 195. Prior to the intervention, no significant group differences were observed in total mindfulness scores [*F*(3,76) = 1.72, *p* = 0.169], indicating baseline equivalence. Results of the mixed-design repeated-measures ANOVA revealed a significant main effect of time [*F*(1,76) = 255.330, *p* < 0.001, η*_p_*^2^ = 0.771], indicating a significant overall increase in mindfulness following the intervention. A significant main effect of group was also observed [*F*(3,76) = 28.771, *p* < 0.001, η*_p_*^2^ = 0.532], suggesting overall differences in mindfulness levels among the training conditions. Moreover, the time × group interaction was significant [*F*(3,76) = 134.173, *p* <0.001, η*_p_*^2^ = 0.842], indicating that changes in mindfulness over time differed significantly across training groups. Simple effects analyses showed that post-intervention mindfulness levels increased significantly in the MT and CT groups (both *p* < 0.001), whereas a slight but significant decrease was observed in the RFB group (*p* = 0.01). Although the control group showed a statistically significant change (*p* < 0.01), the effect size was small relative to the intervention groups. Exploratory analyses suggested that increases in mindfulness were mainly attributable to gains in the Acting with Awareness and Non-judging dimensions. These results support the positive regulatory effects of MT and CT on mindfulness levels in police cadets.

### 3.3 Effects of training interventions on shooting performance in police cadets

After the intervention, all three experimental groups showed significant improvements from pre-test to post-test in SST, RST, TST, and total shooting scores (all *p* < 0.001), indicating that all three training modalities effectively enhanced shooting performance in police cadets (Table 4). Among them, the CT group exhibited the greatest improvements across all sub-tasks and in total scores. In the control group, a small but significant increase was observed in total shooting scores after the intervention (*p* < 0.05), whereas no significant pre–post changes were found in static, rapid, or tactical shooting (*p* = 0.425, 0.267, and 0.070, respectively).

**Figure 5:**
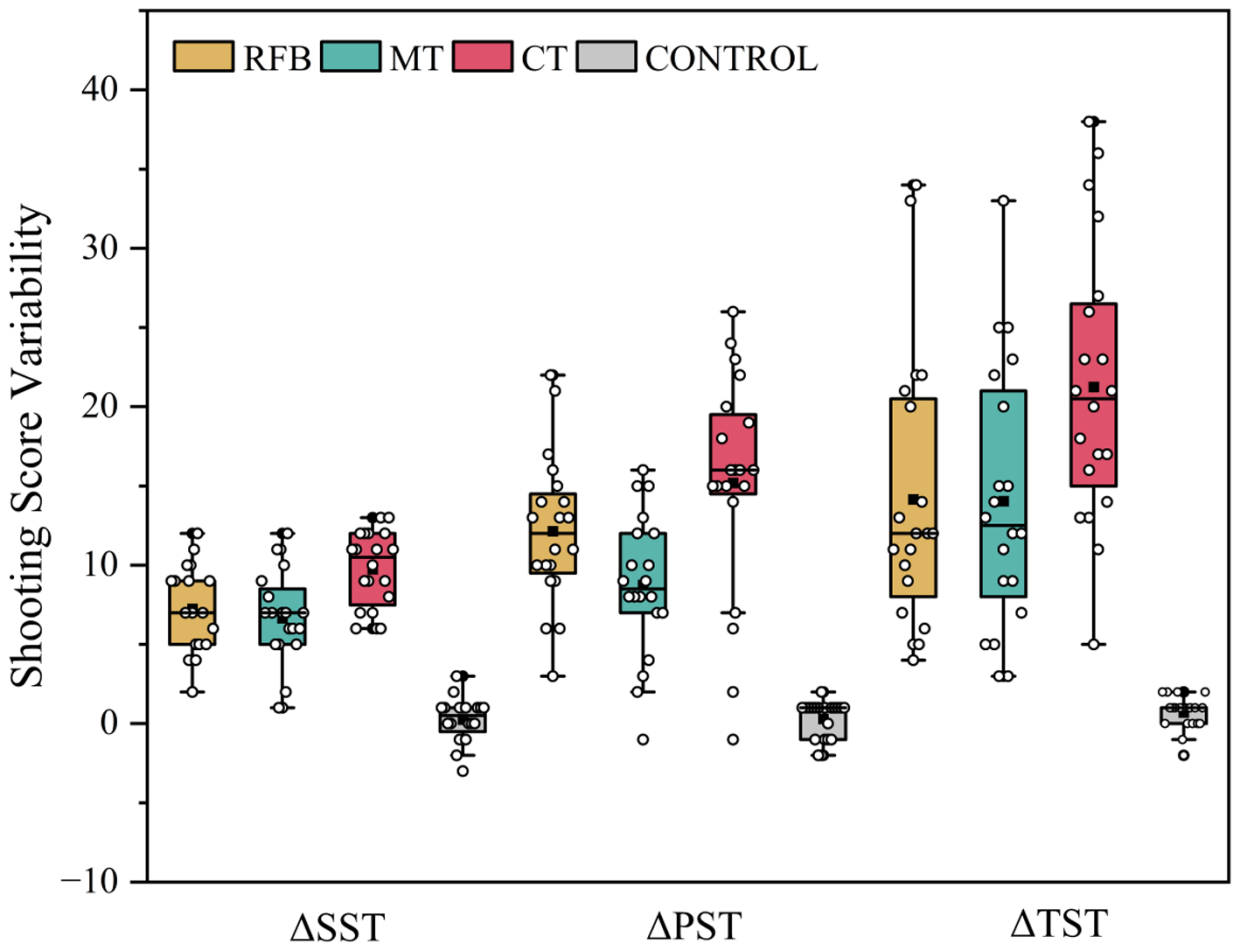
Distribution of shooting score changes (Δ) across intervention groups. Distribution of changes in shooting scores (post−pre) for static (ΔSST), rapid (ΔPST), and tactical (ΔTST) shooting tasks across intervention groups. Boxes indicate the interquartile range with medians shown as horizontal lines; whiskers represent 1.5 × IQR. Group means are marked by black squares, individual observations by white dots, and outliers by black hexagons.

As shown in Figure 5, improvements in static, rapid, and tactical shooting were greater in all experimental groups than in the control group, with the largest gains observed in the CT group. Welch ANOVA revealed significant between-group differences in shooting performance improvements (Δ) across all three shooting tasks. Significant group effects were observed for ΔSST [*F*(3, 76) = 54.588, *p* < 0.001, η*_p_*^2^ = 0.685], ΔPST [*F*(3, 76) = 35.523, *p* <0.001, η*_p_*^2^ = 0.585], ΔTST [*F*(3, 76) = 26.528, *p* <0.001, η*_p_*^2^ = 0.518].

*Post hoc* Games-Howell comparisons indicated significant between-group differences in shooting performance improvements across tasks. In the SST, improvements in the CT group were significantly greater than those in the RFB (*p* = 0.016) and MT groups (*p* = 0.006), with no difference between the RFB and MT groups (*p* = 0.910). In the RST, the CT group showed greater improvements than the MT group (*p* = 0.008), whereas differences between the RFB group and both the MT (*p* = 0.108) and CT groups (*p* = 0.388) were not significant. In the TST, the CT group exhibited a non-significant trend toward greater improvement compared with the RFB (*p* = 0.065) and MT groups (*p* = 0.053). Across all three tasks, improvements in all experimental groups were consistently greater than those in the control group (all *p* < 0.001).

## 4 Discussion

With respect to Hypothesis 1 (H1), the results indicate that, relative to the control group, RFB, MT, and CT all enhanced autonomic regulation following the intervention, reflected in reduced HR and systematic shifts in frequency-domain HRV indices. The strongest effects were observed in the CT group, supporting H1. A reduction in HR generally indicates lower physiological arousal and is commonly associated with increased vagal modulation. Coordinated changes in HRV spectral measures suggest improvement in overall autonomic regulatory state. In this study, LF, HF, and the LF/HF ratio were not interpreted as markers of specific autonomic branches, but as indicators of global HRV level and regulatory pattern changes, consistent with previous findings (Schneider et al., 2025; Zimatore et al., 2026). From a sport psychology perspective, enhanced autonomic regulation reflects not only reduced baseline arousal but also greater physiological stability and recovery under stress. Both regarded as core psychophysiological foundations of high-pressure performance. These findings align with evidence that RFB strengthens vagal tone through cardiorespiratory coupling (Lehrer et al., 2020; Wang et al., 2024) and that mindfulness interventions reduce baseline arousal while facilitating physiological recovery (Hooi et al., 2025; Williams et al., 2023). Long-term autonomic training may further promote regulatory flexibility across task demands (Mesnage et al., 2025), which is consistent with the superior physiological outcomes seen in the CT group. Although some HRV indices changed significantly in the control group, the pattern lacked directional consistency or functional specificity. This suggests that routine training or time-related factors may produce fluctuations without stable regulatory gains. The link between HRV and subjective psychological states may weaken under chronic occupational stress (Schilling et al., 2020). It should be interpreted alongside task context and behavioral performance. Overall, by jointly targeting respiratory and psychological regulation, CT appears particularly effective in strengthening autonomic flexibility under pressure.

With respect to Hypothesis 2 (H2), mindfulness increased markedly in the MT and CT groups relative to the control group (both *p* < 0.001), whereas the RFB group showed a small but significant decrease (*p* < 0.05). This pattern remains consistent with H2, which predicted that the direct effect of RFB on mindfulness would be limited compared with interventions explicitly targeting attentional awareness. Through structured attention training and nonjudgmental awareness, MT enhances present-moment awareness and emotion regulation capacity (Kabat-Zinn, 2023; Baer et al., 2006; Jha et al., 2010; Tang et al., 2015). These mechanisms produce stable psychological benefits in police training contexts. The CT group demonstrated greater overall gains than the MT-only group, suggesting that physiological regulation may create favorable conditions for the development and maintenance of mindfulness awareness. Previous research has indicated that enhanced autonomic regulation supports attentional stability and may strengthen MT effects (Faller et al., 2019; Brown et al., 2021; Mesnage et al., 2025). In contrast, RFB primarily targets bottom-up physiological rhythm regulation rather than attentional or metacognitive processes (Lalanza et al., 2023). This likely explains its limited influence on mindfulness outcomes and aligns with the theoretical expectation of H2.

With respect to Hypothesis 3 (H3), all three interventions significantly improved SST, RST, and TST performance relative to the control group, supporting the facilitating role of psychological skills training in shooting tasks. Although the control group showed a slight increase in total score, this did not translate into stable task-specific improvements. This suggests that routine instruction alone has limited cross-context effects. Training benefits varied by task complexity, with the CT group showing clear advantages in high-load conditions such as the RST and TST, consistent with the context-dependent prediction of H3. SST places high demands on fine motor control and sustained attention, and the uniform gains observed across experimental groups align with the classical view that moderate arousal reduction supports stable motor execution (Yerkes and Dodson, 1908; Hong et al., 2022). By contrast, the RST and TST impose greater time pressure, postural transitions, and situational uncertainty. These factors increase demands on executive control, emotion regulation, and decision efficiency. Performance in such complex tasks depends not only on technical proficiency but heavily on attentional control, emotional stability, and decision-making efficiency (Englert et al., 2021; Zhou et al., 2025). This provides a theoretical basis for the superior CT effects. Overall, CT produced the strongest performance gains, particularly under high time pressure and situational complexity. These results indicate that the performance-enhancing effects of psychological skills training are task dependent, with integrated interventions being especially effective when perceptual-motor, temporal, and emotional demands rise simultaneously.

Taken together, the three hypotheses suggest that different psychological skills training approaches operate through partially distinct yet complementary psychophysiological and cognitive pathways. RFB primarily reduces physiological arousal through improved autonomic regulation, whereas MT more directly targets attentional awareness and psychological regulation. By integrating these mechanisms, CT generates a synergistic effect and shows superior performance under high situational complexity. This pattern is consistent with policing and military research, which indicates that improvements in complex task performance often depend on the coordinated optimization of multilevel regulatory mechanisms (Spiegel and Sutter, 2025). Operational shooting tasks typically involve dynamic posture shifts and situational uncertainty. Evidence suggests that single-dimension training cannot fully address these multidimensional demands (Talarico et al., 2023; Rodriguez et al., 2024). Together, these findings support the contextual and theoretical value of integrated CT models in police firearms training.

### 4.1 Theoretical implications and practical applications

At the theoretical level, this randomized controlled study simultaneously compared physiologically oriented, psychologically oriented, and integrated interventions. It provides empirical evidence for the synergistic contribution of autonomic regulation and attention–emotion regulation to high-pressure motor performance. The findings extend brain–heart coupling frameworks into policing and quasi-military settings. By incorporating multi-context shooting tasks, the study also demonstrates the context dependence of training effects and avoids conclusions based solely on low-complexity performance conditions. At the practical level, the results indicate that integrating psychological skills training with routine firearms instruction is both feasible and advantageous. Compared with single-modality approaches, CT appears particularly suited to performance demands involving high time pressure and situational uncertainty. Training programs may therefore be structured hierarchically according to developmental stage and task demands to optimize training efficiency.

### 4.2 Limitations and directions for future research

While providing empirical support for psychological skills training in police shooting, the study is limited by its small sample. First, it may affect the stability of results and generalizability, highlighting the need for larger or multicenter replication. Second, the present research focused on short-term intervention effects; the long-term maintenance of training outcomes and their transfer to real-world law enforcement contexts require further longitudinal investigation. Third, a mindfulness scale with contextualized wording adjustments was used, while retaining the original structure and dimensions. Although content validity was ensured through expert review and good internal consistency was observed in both the pilot and formal samples, the small pilot sample precluded a systematic examination of structural validity. Future studies should therefore conduct exploratory and confirmatory factor analyses with larger samples to verify structural stability and measurement equivalence. Finally, as shooting performance and HRV indices were the primary outcome variables, future research may incorporate more fine-grained cognitive or neurophysiological measures. This would further elucidate the mechanisms underlying CT effects and enhance the applicability of the findings to police training practice.

Taken together, these findings highlight the overall effectiveness of the interventions and inform the conclusions of the present study.

## 5 Conclusion

This randomized controlled study examined the effects of RFB, MT, and CT on autonomic regulation, mindfulness, and multi-context pistol shooting in police cadets. All three interventions improved psychophysiological regulation and shooting performance, with CT producing the strongest overall effects, particularly in high-complexity tasks. These results suggest that physiologically and psychologically oriented training can operate synergistically under high-pressure conditions, supporting complex task execution. Empirically, the findings reinforce the psychophysiological synergistic regulation framework and provide a basis for systematic, tiered psychological skills training in police shooting.

## Data Availability

The original contributions presented in the study are included in the article/supplementary material, further inquiries can be directed to the corresponding author.

## References

[ref1] Amekran Y. Damoun N. El Hangouche A. J. (2024). Analysis of frequency-domain heart rate variability using absolute versus normalized values: implications and practical concerns. Front. Physiol. 15:1470684. doi: 10.3389/fphys.2024.1470684, 39345784 PMC11427882

[ref2] Baer R. A. Smith G. T. Hopkins J. Krietemeyer J. Toney L. (2006). Using self-report assessment methods to explore facets of mindfulness. Assessment 13, 27–45. doi: 10.1177/1073191105283504, 16443717

[ref3] Bahameish M. Stockman T. (2024). Short-term effects of heart rate variability biofeedback on working memory. Appl. Psychophysiol. Biofeedback 49, 219–231. doi: 10.1007/s10484-024-09624-7, 38366274 PMC11101506

[ref4] Besson C. Baggish A. L. Monteventi P. Schmitt L. Stucky F. Gremeaux V. (2025). Assessing the clinical reliability of short-term heart rate variability: insights from controlled dual-environment and dual-position measurements. Sci. Rep. 15:5611. doi: 10.1038/s41598-025-89892-3, 39955401 PMC11829968

[ref5] Brown L. A. Rando A. A. Eichel K. Van Dam N. T. Celano C. M. Huffman J. C. . (2021). The effects of mindfulness and meditation on vagally mediated heart rate variability: a meta-analysis. Psychosom. Med. 83, 631–640. doi: 10.1097/PSY.0000000000000900, 33395216 PMC8243562

[ref6] Englert C. Dziuba A. Bertrams A. Martarelli C. (2021). An investigation of the effects of self-reported self-control strength on shooting performance. Psychol. Sport Exerc. 52:101839. doi: 10.1016/j.psychsport.2020.101839

[ref7] Faller J. Cummings J. Sajda P. (2019). Regulation of arousal via online neurofeedback improves human performance in a demanding sensory-motor task. Proc. Natl. Acad. Sci. 116, 7598–7603. doi: 10.1073/pnas.1904484116, 30862731 PMC6442591

[ref8] Faul F. Erdfelder E. Lang A.-G. Buchner A. (2007). G*power 3: a flexible statistical power analysis program for the social, behavioral, and biomedical sciences. Behav. Res. Methods 39, 175–191. doi: 10.3758/BF03193146, 17695343

[ref9] Göçmen R. Aktop A. Pınar Y. Toktaş N. Jandačková V. K. (2023). The effect of heart rate variability biofeedback on basketball performance tests. Appl. Psychophysiol. Biofeedback 48, 461–470. doi: 10.1007/s10484-023-09600-7, 37490184

[ref10] Hong X. Xu A. Shi Y. Geng L. Zou R. Guo Y. (2022). The effect of red and blue on gross and fine motor tasks: confirming the inverted-U hypothesis. Front. Psychol. 12:744913. doi: 10.3389/fpsyg.2021.744913, 35069324 PMC8770271

[ref11] Hooi L. Chen P. Tan K. Vries M. Wong H. (2025). Effects of mindfulness breathing meditation on stress and cognitive functions: a heart rate variability and eye-tracking study. Sci. Rep. 15:37185. doi: 10.1038/s41598-025-23727-z, 41136540 PMC12552627

[ref12] Huber A. Koenig J. Bruns B. Bendszus M. Friederich H.-C. Simon J. J. (2025). Brain activation and heart rate variability as markers of autonomic function under stress. Sci. Rep. 15:28114. doi: 10.1038/s41598-025-12430-8, 40751053 PMC12316975

[ref13] Jha A. P. Izaguirre M. K. Adler A. B. (2025). Mindfulness training in military settings: emerging evidence and best-practice guidance. Curr. Psychiatry Rep. 27, 393–407. doi: 10.1007/s11920-025-01608-6, 40456956 PMC12162711

[ref14] Jha A. P. Stanley E. A. Kiyonaga A. Wong L. Gelfand L. (2010). Examining the protective effects of mindfulness training on working memory capacity and affective experience. Emotion 10, 54–64. doi: 10.1037/a0018438, 20141302

[ref15] Kabat-Zinn J. (2023). Wherever You Go, There You Are: Mindfulness Meditation in Everyday Life. UK: Hachette UK.

[ref16] Kabat-Zinn J. (2003). Mindfulness-based interventions in context: past, present, and future. Clin. Psychol. Sci. Pract. 10, 144–156. doi: 10.1093/clipsy/bpg016

[ref17] Lalanza J. F. Lorente S. Bullich R. García C. Losilla J.-M. Capdevila L. (2023). Methods for heart rate variability biofeedback (HRVB): a systematic review and guidelines. Appl. Psychophysiol. Biofeedback 48, 275–297. doi: 10.1007/s10484-023-09582-6, 36917418 PMC10412682

[ref18] Lee S. Kim J. H. Kim H. Kim H. Kim S. H. Park S. S. . (2025). Investigating the effect of mindfulness training for stress management in military training: the relationship between the autonomic nervous system and emotional regulation. BMC Psychol. 13:13. doi: 10.1186/s40359-024-02322-3, 39773484 PMC11706002

[ref19] Leganes-Fonteneau M. Bates M. E. Muzumdar N. Pawlak A. Islam S. Vaschillo E. . (2021). Cardiovascular mechanisms of interoceptive awareness: effects of resonance breathing. Int. J. Psychophysiol. 169, 71–87. doi: 10.1016/j.ijpsycho.2021.09.003, 34534600 PMC13430568

[ref20] Lehrer P. M. Gevirtz R. (2014). Heart rate variability biofeedback: how and why does it work? Front. Psychol. 5:756. doi: 10.3389/fpsyg.2014.00756, 25101026 PMC4104929

[ref21] Lehrer P. Kaur K. Sharma A. Shah K. Huseby R. Bhavsar J. . (2020). Heart rate variability biofeedback improves emotional and physical health and performance: a systematic review and meta-analysis. Appl. Psychophysiol. Biofeedback 45, 109–129. doi: 10.1007/s10484-020-09466-z, 32385728

[ref22] Lehrer P. M. Vaschillo E. Vaschillo B. (2000). Resonant frequency biofeedback training to increase cardiac variability: rationale and manual for training. Appl. Psychophysiol. Biofeedback 25, 177–191. doi: 10.1023/A:1009554825745, 10999236

[ref23] Li H. Chen X. Huang C. Du W. (2026). Effects of acute high-altitude exposure on heart rate variability: a systematic review and meta-analysis. Front. Physiol. 16:1696346. doi: 10.3389/fphys.2025.1696346, 41561154 PMC12812737

[ref24] Li H. Yang Q. Wang B. (2025). Effects of psychological interventions on anxiety in athletes: a meta-analysis based on controlled trials. Front. Psychol. 16:1621635. doi: 10.3389/fpsyg.2025.1621635, 40851578 PMC12368976

[ref25] Liu Y. Hou Y. Quan H. Zhao D. Zhao J. Cao B. . (2023). Mindfulness training improves attention: evidence from behavioral and event-related potential analyses. Brain Topogr. 36, 243–254. doi: 10.1007/s10548-023-00938-z, 36697933

[ref26] Meng Y. Mao K. Li C. (2020). Validation of a short-form five facet mindfulness questionnaire instrument in China. Front. Psychol. 10:3031. doi: 10.3389/fpsyg.2019.03031, 32010036 PMC6978791

[ref27] Mesnage R. Holley A. Grundler F. Martinez-Tellez B. de Wilhelmi Toledo F. Croisille P. (2025). Long-term fasting-induced parasympathetic and sympathetic autonomic nervous system modulation in a subgroup of the GENESIS study. Int. J. Obes. 49, 2125–2128. doi: 10.1038/s41366-025-01843-0, 40634681 PMC12532600

[ref28] Prakash R. S. (2021). Mindfulness meditation: impact on attentional control and emotion dysregulation. Arch. Clin. Neuropsychol. 36, 1283–1290. doi: 10.1093/arclin/acab053, 34651648 PMC8517620

[ref29] Quigley K. S. Gianaros P. J. Norman G. J. Jennings J. R. Berntson G. G. de Geus E. J. C. (2024). Publication guidelines for human heart rate and heart rate variability studies in psychophysiology-part 1: physiological underpinnings and foundations of measurement. Psychophysiology 61:e14604. doi: 10.1111/psyp.14604, 38873876 PMC11539922

[ref30] Reinebo G. Alfonsson S. Jansson-Fröjmark M. Rozental A. Lundgren T. (2024). Effects of psychological interventions to enhance athletic performance: a systematic review and meta-analysis. Sports Med. 54, 347–373. doi: 10.1007/s40279-023-01931-z, 37812334 PMC10933186

[ref31] Rodriguez J. Fredella K. Labhart J. Bunn J. A. Wagner M. (2024). Relationships between physical training and marksmanship performance in tactical law enforcement officers. Policing Int. J. 47, 1111–1125. doi: 10.1108/PIJPSM-11-2023-0148

[ref32] Sakakibara M. Kaneda M. Oikawa L. O. (2020). Efficacy of paced breathing at the low-frequency peak on heart rate variability and Baroreflex sensitivity. Appl. Psychophysiol. Biofeedback 45, 31–37. doi: 10.1007/s10484-019-09453-z, 31781925

[ref33] Schilling R. Herrmann C. Ludyga S. Colledge F. Brand S. Pühse U. . (2020). Does cardiorespiratory fitness buffer stress reactivity and stress recovery in police officers? A real-life study. Front. Psych. 11:594. doi: 10.3389/fpsyt.2020.00594, 32670116 PMC7331850

[ref34] Schneider M. Rominger C. Schwerdtfeger A. R. (2025). Associations between positive affect and heart rate variability: a systematic review. Curr. Cardiol. Rep. 27:148. doi: 10.1007/s11886-025-02299-4, 41128836 PMC12549754

[ref35] Simas V. Schram B. Canetti E. F. D. Maupin D. Orr R. (2022). Factors influencing marksmanship in police officers: a narrative review. Int. J. Environ. Res. Public Health 19:14236. doi: 10.3390/ijerph192114236, 36361117 PMC9655518

[ref36] Spalding D. M. Ejoor T. Zhao X. Bomarsi D. Ciliberti M. Ottaviani C. . (2025). Effects of a brief resonance frequency breathing exercise on heart rate variability and inhibitory control in the context of generalized anxiety disorder. Appl. Psychophysiol. Biofeedback 50, 213–233. doi: 10.1007/s10484-025-09687-0, 39924637 PMC12081530

[ref37] Sparacio A. IJzerman H. Ropovik I. Giorgini F. Spiessens C. Uchino B. N. . (2024). Self-administered mindfulness interventions reduce stress in a large, randomized controlled multi-site study. Nat. Hum. Behav. 8, 1716–1725. doi: 10.1038/s41562-024-01907-7, 38862815 PMC11420060

[ref38] Spiegel C. B. Sutter C. (2025). Training and transfer of executive functions for police- and military-related tasks. J. Police Crim. Psychol. doi: 10.1007/s11896-025-09789-9

[ref39] Talarico M. K. Morelli F. Yang J. Chaudhari A. Onate J. A. (2023). Estimating marksmanship performance during walking while maintaining weapon aim. Appl. Ergon. 113:104096. doi: 10.1016/j.apergo.2023.104096, 37490790

[ref40] Tang Y. Y. Hölzel B. K. Posner M. I. (2015). The neuroscience of mindfulness meditation. Nat. Rev. Neurosci. 16, 213–225. doi: 10.1038/nrn3916, 25783612

[ref41] Tarvainen M. P. Niskanen J.-P. Lipponen J. A. Ranta-Aho P. O. Karjalainen P. A. (2014). Kubios HRV – heart rate variability analysis software. Comput. Methods Prog. Biomed. 113, 210–220. doi: 10.1016/j.cmpb.2013.07.024, 24054542

[ref42] Wang Y. Lei S.-M. Fan J. (2023). Effects of mindfulness-based interventions on promoting athletic performance and related factors among athletes: a systematic review and meta-analysis of randomized controlled trials. Int. J. Environ. Res. Public Health 20:2038. doi: 10.3390/ijerph20032038, 36767403 PMC9915077

[ref43] Wang Y.-L. Wu W.-X. Yang C.-C. Huang S.-M. Chang C.-C. Li C.-R. . (2024). Heart rate variability biofeedback enhances cognitive, motor, psychological, and autonomic functions in post-stroke rehabilitation. Int. J. Psychophysiol. 203:112411. doi: 10.1016/j.ijpsycho.2024.112411, 39116804

[ref44] Williams M. Honan C. Skromanis S. Sanderson B. Matthews A. J. (2023). Psychological and attentional outcomes following acute mindfulness induction among high anxiety individuals: a systematic review and meta-analysis. J. Psychiatr. Res. 159, 334–346. doi: 10.1016/j.jpsychires.2023.01.023 38215647

[ref45] Yerkes R. M. Dodson J. D. (1908). The relation of strength of stimulus to rapidity of habit-formation. J. Comp. Neurol. Psychol. 18, 459–482. doi: 10.1002/cne.920180503

[ref46] Zhao C. Wang K. Li D. Li Y. Wang Z. Liu Y. (2024). Relationship between state anxiety, heart rate variability, and shooting performance in adolescent shooters. BMC Psychol. 12:736. doi: 10.1186/s40359-024-02062-4, 39695791 PMC11658281

[ref47] Zhou L. Liu H. Su H. (2025). Effect of emotional regulation on performance of shooters during competition: an ecological momentary assessment study. PLoS One 20:e0318872. doi: 10.1371/journal.pone.0318872, 40131872 PMC11936280

[ref48] Zimatore G. Russo S. Gallotta M. C. Passalacqua G. Zaborova V. Campanella M. . (2026). HRV in stress monitoring by AI: a scoping review. Appl. Sci. 16:23. doi: 10.3390/app16010023

